# Total ear canal ablation for the treatment of auricular hemangiosarcoma in a cat: case report

**DOI:** 10.29374/2527-2179.bjvm012626

**Published:** 2026-09-22

**Authors:** Júlia Vaz Feio, Elaine Lopes de Carvalho

**Affiliations:** 1 Programa de Pós-Graduação em Saúde e Produção Animal na Amazônia, Instituto de Saúde e Produção Animal, Universidade Federal Rural da Amazônia (UFRA), Belém, PA, Brazil; 2 Clínica Veterinária Amor e Patas, Ananindeua, PA, Brazil; 3 Laboratório de Histologia e Embriologia Animal (LHEA), UFRA, Belém, PA, Brazil

**Keywords:** ear neoplasia, total ablation, cryosurgery, hemangiosarcoma, histopathology, neoplasia auricular, ablação total, criocirurgia, hemangiossarcoma, histopatologia

## Abstract

Ear neoplasms are growths that commonly affect light-coated, unpigmented felines exposed to prolonged sunlight. Another factor associated with these growths is the constant tissue trauma caused by intense pruritus in cases of chronic otitis. Any tissue making up the structure of the pinna can undergo changes and initiate pathogenic cell proliferation. In this context, cutaneous hemangiosarcomas may arise from alterations in the ear's vascular endothelium, albeit less frequently. This report describes the case of a 12-year-old, white, mixed-breed cat with a history of prior pinnectomy and local recurrence. To treat the new lesion, total ear canal ablation combined with cryotherapy of the surgical bed was selected to maximize recurrence-free survival. Histopathological analysis of the surgical specimen confirmed a moderate grade hemangiosarcoma associated with chronic otitis, with clear margins. The patient was referred to for oncological follow-up and remains free of recurrence one year after the procedure.

## Resumo

Neoplasias auriculares são proliferações teciduais que frequentemente acometem felinos de pelagem clara e despigmentada expostos à radiação solar prolongada. Outro fator associado a essas lesões é o trauma tecidual constante, decorrente de prurido intenso em casos de otite crônica. Qualquer tecido constituinte do pavilhão auricular pode sofrer alterações e desencadear uma proliferação celular patogênica. Nesse contexto, hemangiossarcomas cutâneos podem surgir a partir de alterações no endotélio vascular da orelha, embora com menor frequência. Este relato descreve o caso de um felino de 12 anos, branco e sem raça definida, com histórico de pinnectomia prévia e recidiva local. Para o tratamento da nova lesão, optou-se pela ablação total do conduto auditivo associada à crioterapia do leito cirúrgico, visando aumentar a sobrevida livre de recidiva. A análise histopatológica da amostra cirúrgica confirmou um hemangiossarcoma de grau moderado associado à otite crônica, com margens livres. O paciente foi encaminhado para acompanhamento oncológico e permanece livre de recidiva um ano após o procedimento.

## Introduction

Cutaneous neoplasms are strongly correlated with prolonged exposure to ultraviolet (UV) rays, acting as a cumulative carcinogenic factor throughout an organism's life (Mauldin & Peters-Kennedy, 2016). Solar radiation exposure is primarily linked to epithelial neoplasms, such as squamous cell carcinomas, basal cell carcinomas, and malignant melanomas. However, there is sufficient evidence linking it to the development of vascular tumors (Parisi et al., 2022).

Hemangiosarcoma (HSA)—a malignant neoplasm arising from growth abnormalities in cells of the endothelial lineage (i.e., blood vessels)—is characterized by its invasive and metastatic nature. It commonly originates in highly vascularized organs, such as the spleen, liver, lungs, heart, and kidneys. Although less frequently reported, cutaneous HSA does occur, either as a primary tumor or resulting from metastasis. In its cutaneous form, it appears most often on the abdominal skin, prepuce, and extremities—areas with sparse hair coverage. Among animal species, dogs are the most affected, with fewer reports in cats (Couto Filho et al., 2022).

Dermal hemangiosarcomas present as dark, firm, raised nodules; subcutaneous forms appear as fluctuant, firm, or soft masses, potentially accompanied by ecchymosis. Intratumoral hemorrhage may create the impression of rapid growth. These tumors have high metastatic potential, frequently spreading to the spleen and heart. Radical surgical excision is the recommended treatment (Rhodes, 2005; Albertus, 2011).

Neoplasms can be diagnosed via cytological examination using fine-needle aspiration, capillary sampling, or impression smear techniques (Carvalho et al., 2019; Tizziani Júnior et al., 2023). An incisional biopsy—in which a fragment of the neoplasm is removed for preliminary histopathological diagnosis (Mello et al., 2021)—may be performed, or alternatively, an excisional biopsy followed by histopathological analysis of the surgical specimen; the latter offers greater safety and speed in removing the potentially cancerous lesion, while also allowing for the assessment of surgical margins free of cellular abnormalities (Braga Filho et al., 2018; Techima et al., 2023).

Surgical treatment is the preferred approach for neoplasms of the pinna and ear canal (Fossum & Caplan, 2021). For neoplasms confined to the pinna, conchectomy is the most widely accepted approach (Lanz & Wood, 2004; Borges et al., 2020). When neoplasms invade the ear canal, vertical ear canal ablation or total ear canal ablation (TECA) are the most recommended and common procedures in global surgical practice, although there are few reports of them in Brazil (Fossum & Caplan, 2021; Techima et al., 2023). Postoperative complications—such as Horner's syndrome, facial nerve paralysis, fistula formation, vestibular disease, hypoglossal nerve dysfunction, and infections—are frequently described in the literature. The TECA technique combined with lateral bulla osteotomy and curettage of the secretory epithelium (LBO) has significantly reduced postoperative complication rates (Lanz & Wood, 2004). Despite the resulting anatomical defect and potential complications, the TECA technique is considered the best option for the surgical resection of infiltrative malignant auricular neoplasms with clear margins, thereby ensuring survival for affected animals (Folk et al., 2022). Cryosurgery is a procedure involving the use of extreme cold to cause selective tissue destruction; it helps minimize the incidence of neoplastic recurrence and can be performed either as a standalone treatment or as an adjunct to conventional surgery, thereby maximizing tumor-free excision margins (De Queiroz et al., 2008). Against this background, this paper reports on the surgical and cryosurgical treatment of a feline patient with a hemangiosarcoma at the base of the ear.

## Case report

A 12-year-old, mixed-breed female cat with a white coat and blue eyes was presented with a growth on the ear pinna; the pinna had previously undergone a pinnectomy due to a neoplasm that had not been identified via histopathology. The patient showed no abnormalities in physiological parameters but was physically thin—despite no changes in dietary habits—and exhibited signs of pain and restlessness, constantly attempting to scratch the lesion.

The neoplastic lesion was actively bleeding heavily, making it impossible to obtain a satisfactory sample for cytological examination (Figure 1). The lesion was cleaned and dressed, and the following medications were prescribed for immediate administration while preoperative tests were conducted: meloxicam (0.05 mg/kg SID), omega-3 (500 mg/animal SID), tranexamic acid (25 mg/kg BID), and gabapentin (10 mg/kg BID). The requested tests included a complete blood count (CBC), serum biochemistry, thoracic radiography, abdominal ultrasonography, echocardiography (Doppler), electrocardiography, and blood pressure measurement.

**Figure 1:**
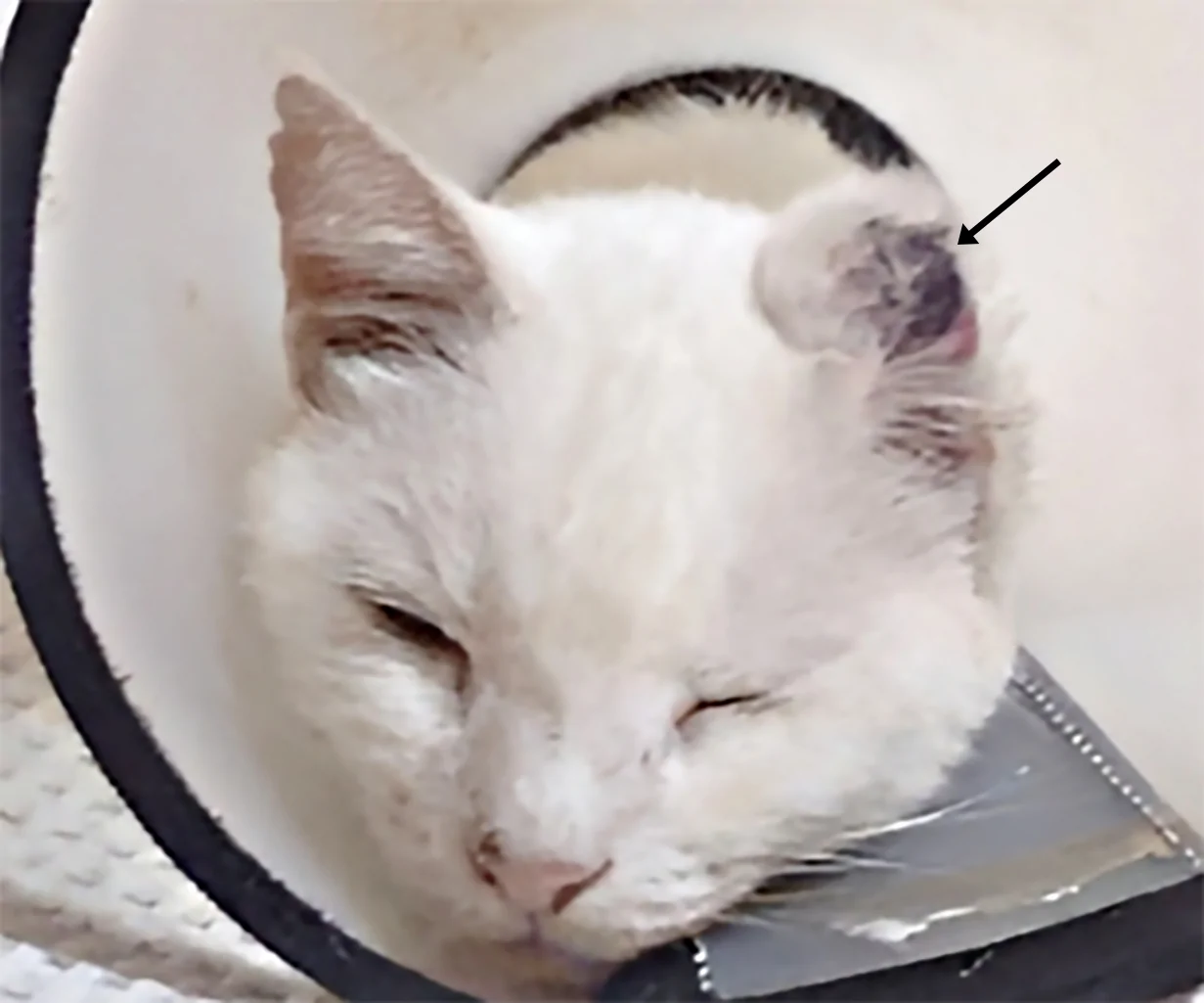
Patient at the initial consultation. Presence of a blackened, friable, and hemorrhagic nodule (arrow) on the left auricle.

The patient's complete blood count revealed severe anemia, as well as inflammation and infection, evidenced by elevated leukocyte counts (Table 1). An intraoperative blood transfusion was recommended; however, due to the owners' financial constraints, they stated they could not purchase the blood unit—which was also unavailable at the local blood bank. To avoid further risks and given the unknown cellular origin of the neoplasm, new medications were prescribed to attempt to correct the anemia prior to the procedure, which was postponed for 14 days. Although supplementation with folic acid, B-complex vitamins, and iron can provide nutrients to oncologic cells, this was the most viable option agreed upon with the owners. During this period, the patient was prescribed cefovecin sodium (8 mg/kg, single dose), norfloxacin (20 mg/kg BID), phytomenadione (5 mg/kg SID), and Eritrós^®^ Cat Paste (1 mL/animal), in addition to continuing gabapentin and omega fatty acids.

**Table 1:** Complete blood count of the feline patient at the initial consultation.

ERYTHROGRAM	
Erythrocytes	2.96 millions/mm^3^
Hemoglobin	5.1 g/dL
Hematocrit	15%
Mean Corpuscular Volume	51 fL
Mean Corpuscular Hemoglobin	17 pg
Mean Corpuscular Hemoglobin Concentration	34 g/dL
Total plasma protein	8 g/dL
Note: anisocytosis ++, polychromasia ++	
LEUKOGRAM	
Total leukocytes	72,230/mm^3^
Rod cells	0/mm^3^
Segmented	67,174/mm^3^
Basophils	0/mm^3^
Eosinophils	722/mm^3^
Typical lymphocytes	3,612/mm^3^
Reactive lymphocytes	0/mm^3^
Monocytes	722/mm^3^
Nucleated erythrocytes	0%
PLATELET PROFILE	
Platelets	155.000/mm^3^
Mean Platelet Volume	9.3 fL
Platelet Distribution Width	15,8

Note: Examination showing an intensely low red series, indicating severe anemia, in addition to severe leukocytosis, indicating the possibility of a septic condition.

No radiographic evidence of pulmonary metastasis was observed (Figure 2); however, the patient presented with *pectus excavatum* and fecal retention, for which a lactulose-based syrup (150 mg/kg BID) and a diet with higher moisture content were prescribed. The owners were unable to proceed with the abdominal ultrasound and echocardiogram to screen for potential metastases in organs such as the spleen, liver, and heart. The other aforementioned preoperative tests showed no abnormalities.

**Figure 2:**
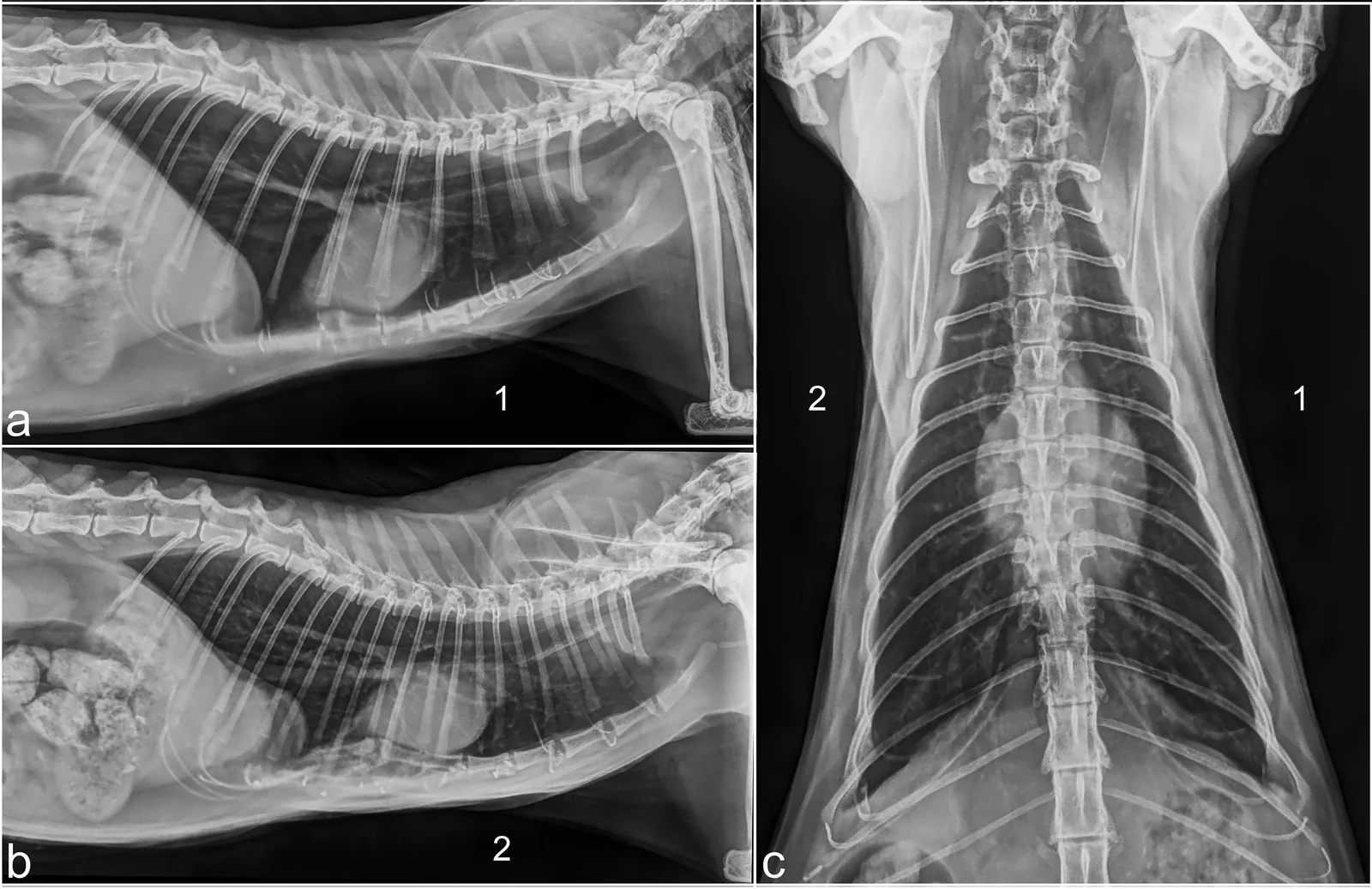
Chest X-ray of the patient free of metastases. (a) left laterolateral projection, (b) right laterolateral projection, (c) ventrodorsal projection. 1 - left side, 2 - right side.

After 10 days, the patient returned for a repeat complete blood count, which showed significant improvement in red and white blood cell counts, although they remained outside the reference ranges (Table 2). Consequently, the patient was cleared to undergo the procedure.

**Table 2:** Complete blood count of the feline patient after pharmacological intervention.

ERYTHROGRAM	
Erythrocytes	4.44 millions/mm^3^
Hemoglobin	7.8 g/dL
Hematocrit	23%
Mean Corpuscular Volume	52 fL
Mean Corpuscular Hemoglobin	18 pg
Mean Corpuscular Hemoglobin Concentration	34 g/dL
Total plasma protein	8.5 g/dL
Note: anisocytosis ++, polychromasia ++	
LEUKOGRAM	
Total leukocytes	66,300/mm^3^
Rod cells	3,978/mm^3^
Segmented	55,029/mm^3^
Basophils	0/mm^3^
Eosinophils	0/mm^3^
Typical lymphocytes	5,304/mm^3^
Reactive lymphocytes	0/mm^3^
Monocytes	1,989/mm^3^
Nucleated erythrocytes	0%
PLATELET PROFILE	
Platelets	232,000/mm^3^
Mean Platelet Volume	11.4 fL
Platelet Distribution Width	16.4

Note: General improvement in the patient's examination after clinical intervention, making her fit for the surgical procedure.

On the day of the procedure, the lesion was heavily contaminated, as the owners had been unable to manage the wound dressings and the patient had been scratching the site. The pre-anesthetic protocol included methadone (0.2 mg/kg) and dexmedetomidine (3 mcg). Induction was performed with propofol (4 mg/kg), and maintenance involved an infusion of remifentanil (15 mcg/kg/h) and ketamine (0.6 mg/kg/h). Subsequently, the area was cleaned and widely clipped (Figure 3a) using chlorhexidine and alcohol. Next, a nerve block of the caudal auricular and auriculotemporal nerves—identified via neurostimulator—was performed, administering bupivacaine (0.3 mg/kg) at each site. Final antisepsis was then carried out, and surgical drapes were placed to begin the procedure (Figure 3b).

**Figure 3:**
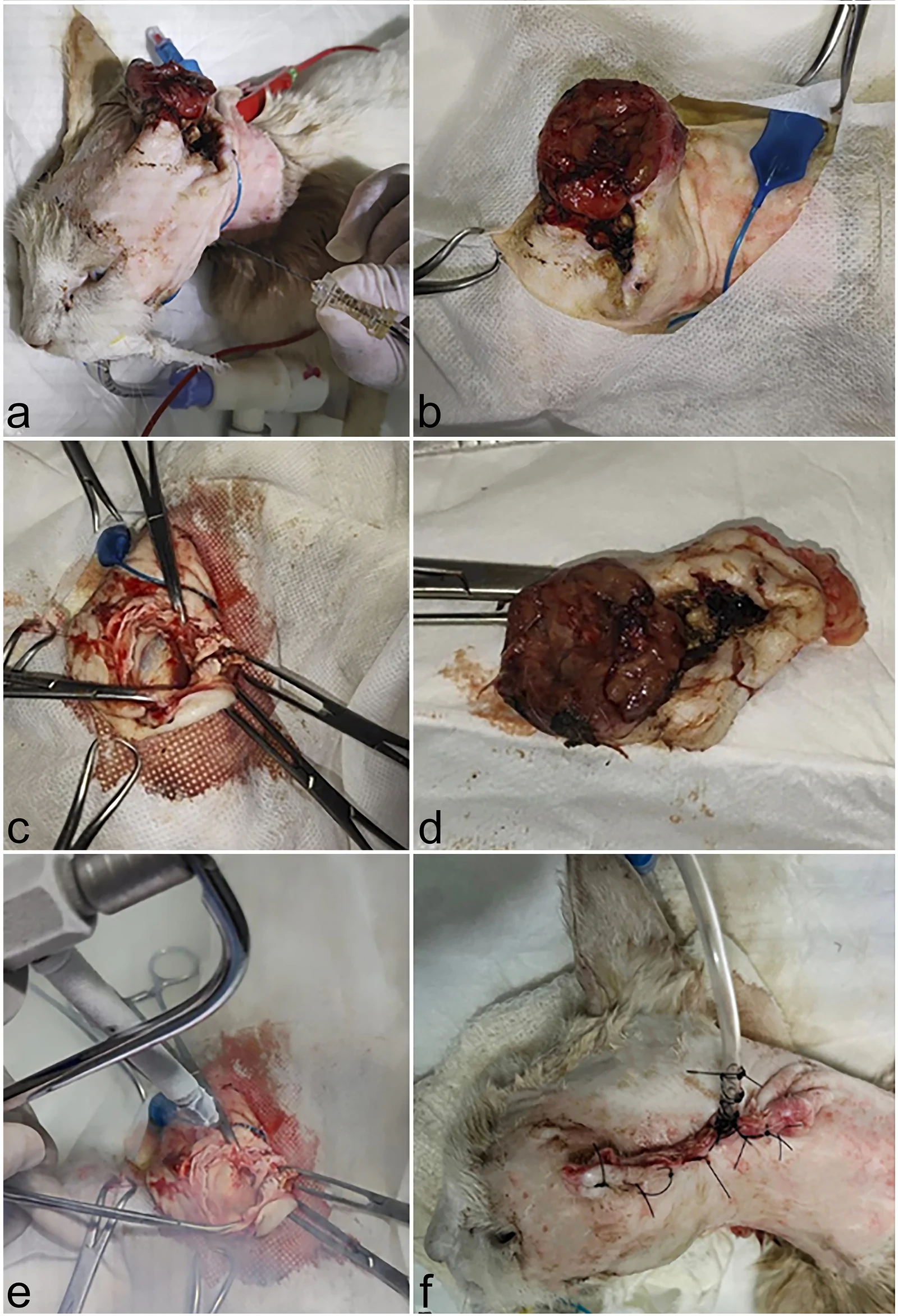
Stages of the surgical procedure and cryosurgery. (a) wide clipping of the area and nerve localization for anesthetic block; (b) surgical site isolated with drapes; (c) surgical defect following en bloc removal; (d) resected surgical specimen; (e) cryosurgery of the surgical bed; (f) closure of the surgical defect and catheter for postoperative bupivacaine adminis.

To begin the procedure, an elliptical incision was made at the base of the ear flap, and tissue dissection was performed around the funnel-shaped cartilages down to the level of the tympanic membrane, while identifying and preserving adjacent nerves and blood vessels. The ear canal was removed en bloc along with the neoplasm and the remaining pinna. Secretory tissue was removed using a curette, followed by lavage with saline solution (Figures 3c and 3d).

To extend the depth margins of the resection, cryosurgery was performed on the surgical bed (Figure 3e) using nitrogen sprays until a thin layer of ice formed on the tissue. The tissue was allowed to rewarm before closure of the surgical defect began. A fenestrated urethral catheter was placed, secured at the depth of the lesion to allow for subsequent local anesthetic administration; muscle and subcutaneous tissues were sutured with 3-0 poliglecaprone using a simple interrupted pattern, and the skin was closed with 3-0 nylon using a Wolf suture pattern (Figure 3f). The surgical specimen was sent for histopathological analysis.

The patient was hospitalized for 24 hours to ensure optimal pain management. Upon immediate recovery from anesthesia, she appeared comfortable and maintained her appetite. However, diminished palpebral reflexes were noted—a risk that had been previously communicated to the animal's owners. The hospitalization protocol included intramuscular methadone (0.2 mg/kg QID), bupivacaine via the catheter (0.3 mg/kg QID), intravenous dexamethasone (0.14 mg/kg SID), intravenous dipyrone (12.5 mg/kg BID), norfloxacin (22 mg/kg BID), maintenance fluid therapy, and Bionew® (0.2 mL/kg). The patient was discharged the following day and returned 48 hours later for catheter removal and re-evaluation.

For home care, the following were prescribed: dipyrone (12.5 mg/kg BID), tramadol hydrochloride (2 mg/kg BID), prednisolone (1 mg/kg SID), palmitoylethanolamide (50 mg/animal SID), and norfloxacin (20 mg/kg BID). Instructions included changing dressings every 24 hours, topically applying a healing ointment containing antibiotics and urea, and using an Elizabethan collar full-time until medical discharge. Additionally, a B-complex vitamin supplement was prescribed to help manage the procedure-induced neuritis that had caused a diminished palpebral reflex. Lubricating eye drops were also used during this period, as the reduced palpebral reflex prevented the spreading of tears across the cornea.

Ten days later, the patient returned for a scheduled re-evaluation; the owners reported that she had managed to remove the Elizabethan collar and dressing, scratching the surgical site until it bled. Examination revealed the loss of some skin sutures, though the subcutaneous tissue showed good healing progress (Figure 4a). Consequently, healing by second intention was indicated for the wound. After 60 days, the wound had completely closed, the patient’s fur had fully regrown (Figures 4b and 4c), and the palpebral reflexes had returned.

**Figure 4:**
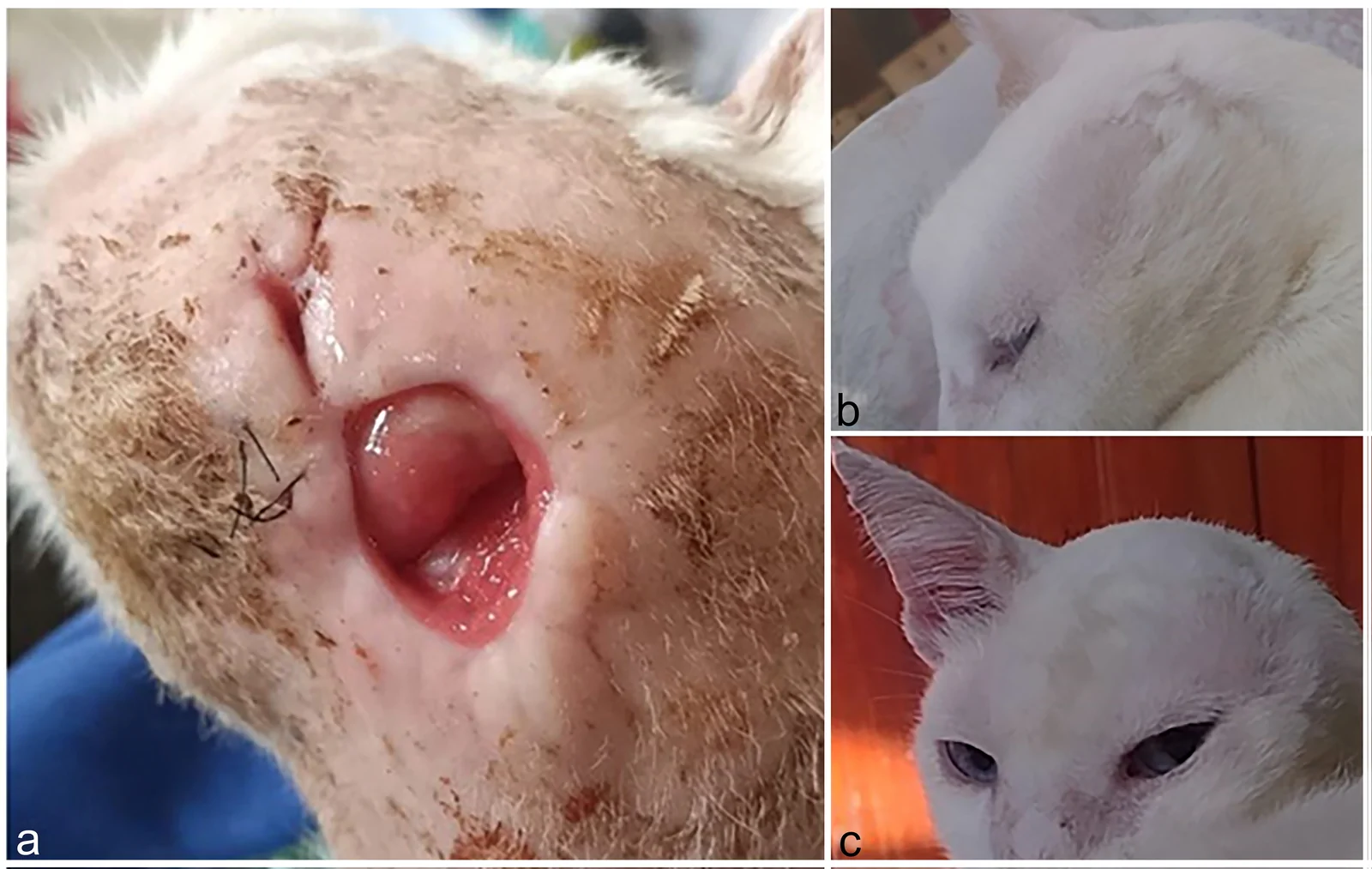
Feline patient in the postoperative period. Healing by second intention (a), complete healing (b, c).

Histopathological analysis was conclusive for intermediate grade hemangiosarcoma associated with marked, diffuse, active chronic otitis. The tissue exhibited a proliferation of spindle-shaped to polygonal cells within the dermis, infiltrating skeletal muscle fibers and forming multiple irregular vascular spaces of varying sizes filled with intraluminal erythrocytes, alongside areas of hemorrhage associated with neoplastic growth (Figure 5). The patient was referred to as oncological follow-up, and in the year following the procedure, she showed no signs of local recurrence or systemic indications of potential metastasis.

**Figure 5:**
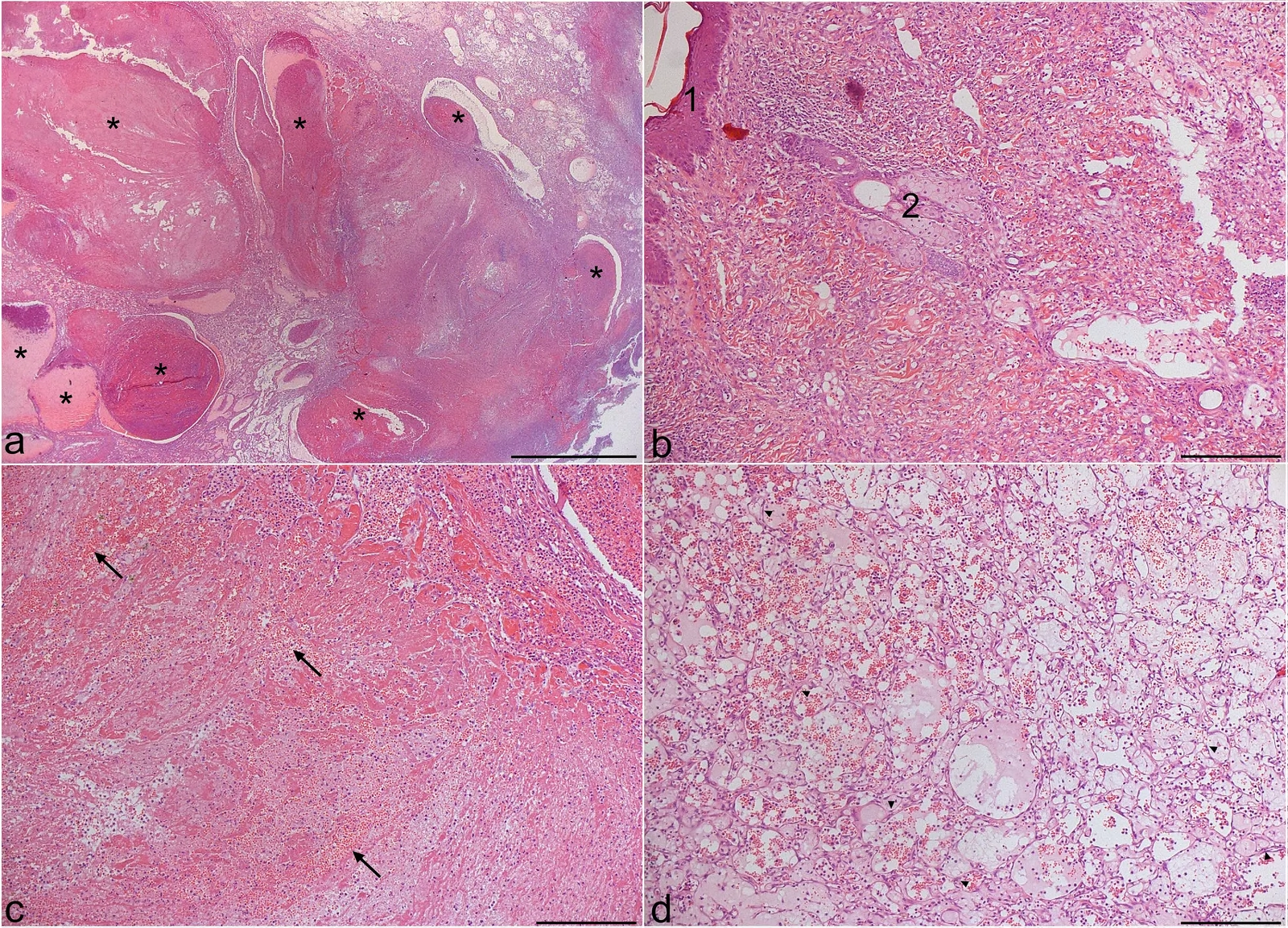
Photomicrograph of the histopathology of the excised auditory canal specimen. (a) Nodular region showing multiple areas of tissue degeneration (*); (b) region near the auricle with altered tissue, showing epidermis (1) and sebaceous gland (2); (c) neoplastic area showing areas of erythrocyte extravasation (arrow); (d) neoplastic area showing endothelial cell hyperplasia (arrowhead). Staining – Hematoxylin and Eosin. Scale bar: a – 2 mm; b, c, and d – 200 µm.

## Discussion

The patient's white coat and age, along with her medical history, contributed to the suspicion of a malignant neoplasm. Just as a lack of coat pigmentation increases ultraviolet ray absorption, advanced age means that a lifetime of sun exposure has a cumulative effect, manifesting as carcinogenic lesions—a common occurrence in elderly cats (Braga Filho et al., 2018; Borges et al., 2020; Parisi et al., 2022).

According to her history, the patient had previously presented with a lesion requiring a pinnectomy; however, no histopathological analysis was performed to assess surgical margins or determine the need for further therapies. Neoplasm recurrence is common following incomplete excision; specifically, cutaneous hemangiosarcoma recurs in 60% of cases, making more radical excision the indicated approach (Albertus, 2011).

Given the lesion's extensive nature, high vascularity, and associated inflammation, it is essential to initiate analgesic, anti-inflammatory, and antioxidant therapy prior to any procedure; this reduces lesion reactivity and ensures a more comfortable postoperative recovery. This multimodal therapy is widely used in oncological patients (Techima et al., 2023). Angiogenesis associated with tumor progression triggers inflammatory factors, even in small, seemingly non-painful lesions (Horta, 2013). The use of omega-3 fatty acids helps inhibit tumorigenesis and cancer dissemination by reducing undesirable cytokines and promoting an anti-cachectic effect, which is beneficial for these patients (Costa, 2017).

Oncology patients frequently present with regenerative anemia (Morris & Dobson, 2001). Anemia is associated with chronic conditions and blood loss. Anemia of chronic disease typically presents as normocytic and normochromic. In contrast, an increase in white blood cell count is more closely associated with the tumor type—being more common in lymphomas and hemangiosarcomas—due to the required hematopoiesis, tissue necrosis, and secondary infections (Albertus, 2011). All these findings were observed in the patient described in this report.

Due to the presence of chronic otitis in the affected ear, along with infection and tumor recurrence, total ear canal ablation was selected as the treatment. The procedure itself carries significant risks of complications, primarily involving damage to the facial nerve and its branches, as well as the potential for abscesses and fistula formation linked to residual secretory epithelial tissue within the tympanic bulla. Consequently, combining the procedure with lateral bulla osteotomy and tissue curettage is recommended (Smeak, 2011). However, due to difficulties performing the bulla osteotomy—possibly caused by bone thickening—that step was omitted; instead, only curettage and lavage of the cavity were performed. No abscesses or fistulas were observed up to one-year post-procedure, the time of this report.

Several patients undergoing this technique have exhibited irreversible neurological signs post-procedure, such as Horner's syndrome—characterized by protrusion of the third eyelid, enophthalmos (deep-set eyeball), miosis (pupil constriction), and upper eyelid ptosis. Head tilt (tilting the head toward the affected side) and drooping of the upper lip on the affected side have been reported (Clarke, 2004; Souza et al., 2005; Mello et al., 2021). Just as the patient in the report by Techima et al. (2023) experienced no complications with the technique, the same outcome was observed in the current case; although a transient loss of the palpebral reflex occurred, it subsequently returned to normal.

Facial nerve paralysis is one of the most common complications following TECA; approximately 56% of cats experience temporary paralysis, and in one-quarter of these cases, it is permanent (Bacon et al., 2003). Temporary paralysis likely results from stretching or retraction of the nerve during surgery and typically resolves within 4 to 6 weeks. During this period, the use of artificial tears is important to lubricate the eye and prevent ulcerative keratitis (Fossum & Caplan, 2021).

Cryosurgery was performed on the surgical bed to achieve a greater depth margin, as surgical excision would have required removing muscle tissue and likely damaging local blood vessels and nerves. It is an easy-to-perform technique, although it requires trained personnel and appropriate equipment (De Queiroz & Matera, 2003). Due to the resistance of cancer cells to freezing, multiple freeze-thaw cycles are performed in the same area to ensure the destruction of the target cells. This technique can be used for small lesions or in locations where obtaining a safe surgical margin is not possible (De Queiroz & Matera, 2003).

Borges et al. (2020) reported a case of hemangiosarcoma on the ear pinna of a female cat—sharing the same phenotypic characteristics as the patient in the current study—which caused an associated aural hematoma; this likely resulted from damage to the vascular endothelial cells, leading to vessel rupture. This demonstrates that, although this tumor type is uncommon in felines, it can occur in any vascularized tissue and should therefore remain a diagnostic consideration during clinical evaluations.

Furthermore, given the conclusive histopathological diagnosis of intermediate grade hemangiosarcoma and the tumor's metastatic nature, lesions in the spleen, liver, and heart were potential risks following total ear canal ablation (Costa, 2017). Due to the patient's emaciation—despite a maintained appetite—an abdominal ultrasound and echocardiogram were requested for further investigation; however, the owner was unable to have these procedures performed. Given the normal cardiac auscultation and absence of abdominal tenderness, combined with the patient's post-surgical survival, the likelihood of metastasis was considered low, although not definitively ruled out. Albertus (2011) notes that, in cases of internal hemangiosarcoma, patient survival is estimated at three months following the procedure.

## Conclusion

Based on this study, it is concluded that cutaneous hemangiosarcoma—despite being less common—should be considered in the differential diagnosis of feline ear neoplasms, as its occurrence is associated with coat characteristics and age. Furthermore, the study reinforces that when dealing with an aggressive neoplasm, veterinarians should recommend surgical procedures that ensure wider clear margins and the use of combined therapies to improve patient survival. The patient in question remains free of recurrence and shows no behavioral changes.
